# How can GBV Research Be Decolonized and Under What Conditions? Critical Reflection on a Comparative Ethnography of Case Studies from Samoa and Peru

**DOI:** 10.1177/10778012251338414

**Published:** 2025-05-05

**Authors:** Jenevieve Mannell

**Affiliations:** 1 Institute for Global Health, University College London, 30 Guilford Street London, UK

**Keywords:** gender-based violence, prevention programming, coloniality, Samoa, Peru

## Abstract

Histories of colonialism matter for research on gender-based violence (GBV) and questions of how to prevent it. This paper aims to contribute to theoretical debates on decolonizing GBV research by reflecting on the challenge of doing so as part of a long-term ethnographic engagement with communities in Samoa and Peru. I present three “epistemic entry-points,” which point to the potential for moving the project of decoloniality forward through increasing the diversity of what we think we know about gender and violence. This presents a radical undertaking for a field currently focused on achieving decolonization through improving leadership from the Global South.

## Background

Colonial histories and their legacy play an important, yet under-recognized, role in the higher rates of gender-based violence (GBV) against women observed in low- and middle-income countries, which overlap considerably with the world's former colonies (Khan et al., 2022). A history of colonialism has been shown to be associated with an increase in the national prevalence of intimate partner violence (as one form of GBV), even when controlling for gross domestic product (GDP) (Brown et al., 2022). Different explanations have been offered for this association by anthropologists, colonial historians, and postcolonial theorists, which I synthesize around three central theoretical propositions.

The first is that the legacy of colonial laws and policies had negative ripple effects for women, systematically undermining their access to land and economic employment in ways that make them more susceptible to violence from men (Peek-Asa et al., 2011). We see this history play out across the African continent, where the development of legal systems by colonial powers often relied on male testimony, placing women at a disadvantage in terms of legal provisions related to marriage and divorce (Sheldon, 2018). In Nigeria, the British Crown implemented policies that disenfranchised women from inheriting land, a practice that may no longer be legal, but persists, reducing women's access to land and increasing their dependency on men (Korieh, 2010). In a similar vein, Margaret Jolly argues for the crucial role played by Christian missionaries in reformulating gender relations in the Pacific through dividing care for family from the “hard” manual labor that women did previously, such as cultivating taro; a gender division of labor further institutionalized in formal schooling of women as part of a Christian education (Jolly, 1989).

Second is that historical trauma of colonialism is passed on intergenerationally, leading to higher levels of aggression and abuse by men against women and gender-diverse individuals (Mohatt et al., 2014). Historical or cultural trauma refers to a disruption in cultural practices that produce a shared meaning or cultural identity for a group with a shared history (Riley et al., 2022). Colonial practices deliberately stripped this identity and existing cultural practices through, for instance, the residential school system imposed on Indigenous peoples in Australia and Canada. The postcolonial theorist and psychiatrist Franz Fanon writes of how this loss of cultural identity creates anger and aggression in the “oppressed,” which may then be internalized and enacted against their own people (Fanon, 2008, 2022). Historical trauma has been widely used to explain the health impacts of systematic oppression, stigmatization, and discrimination experienced by Indigenous populations, for instance in Hawaii and New Zealand (Kaholokula et al., 2020; Koziol-McLain et al., 2007; Riley et al., 2022). It has also been used as a framework for understanding the higher rates of GBV seen in Indigenous communities in comparison to settler populations (Burnette & Figley, 2017).

Third, is Maria Lugones’ concept of the “coloniality of gender” building on Quijano's (2000) original concept of the “coloniality of power.” Lugones (2007) argues that gender relations were significantly transformed by colonial authorities in ways that reinforce men's perpetration of GBV today. She draws on feminist postcolonial scholars, including Oyewumi (2004) and Allen (1984), to show how the colonial project relied on a binary distinction between men and women to design new laws according to colonial ideas, essentially creating a binary concept of gender with clear hierarchies between men and women, which may have been different to societal gender roles pre-colonization.

Each of these theoretical ideas has its relevance for understanding why some countries have such a high prevalence of GBV. Tracing a country's legal statutes and policy documents provides insight into the prioritization of men's needs over women in certain colonial contexts, as shown in Jennie Gamlin's brilliant portrayal of men's control over childbirth practices in Mexico (Gamlin, 2020). Intergenerational or historical trauma has provided a lens for exploring the implications of colonial violence and how this gets passed down through poor mental health outcomes for subsequent generations (Ahmad et al., 2022). The coloniality of gender has provided a feminist approach to studies of colonialism that puts the onus on history and the social structures that have created gender, rather than the widely accepted culprit—culture (Descola & Sahlins, 2013).

However, this literature and its theoretical insights rarely gets taken up into the practice of preventing GBV in high prevalence settings (Mannell et al., 2021). A recent review of the literature on decolonizing GBV research and programming makes important calls for bottom up, community-driven approaches to GBV prevention (Lokot et al., 2024). However, while decolonial theory is often used to explain or rationalize the higher rates of GBV experienced by Indigenous or minority ethnic communities (García-Del Moral, 2018; Hardin, 2002; Jenkins, 2021), it is rarely seen as a concept that can actually be dismantled or deconstructed through GBV programs and interventions. This paper aims to fil this gap by making a theoretical contribution to the decolonial literature through critical reflection on how to achieve *decoloniality* (as a political and epistemic project) in praxis and some of the challenges inherent in this.

I start by describing the process we, as a team, have been through over the past five years in working with Indigenous communities in Samoa and Peru. As part of the Evidence for Violence Prevention in the Extreme (EVE) Project, we have worked toward developing a participatory community-led intervention to reduce GBV, in which we explicitly tried to decolonize GBV research practice as a key aim (Mannell et al., 2021). As part of this process, we have faced several challenges (and experienced some rather unexpected successes), which I summarize. I then reflect on what these challenges taught us about the praxis of GBV prevention from a theoretical standpoint, highlighting three “epistemic entry points” to help further current debates on decolonizing GBV research.

My role in this space is as both an ally and an interlocutor from the rather privileged position of being a white scholar, born and raised in Canada and currently employed by a leading UK university. Over the past 14 years working in GBV prevention research, I have had the honor of learning from Indigenous scholars who were willing to share their worldview with me. While this does not make me even close to an expert in Indigenous anything, it has raised a number of questions for my own GBV research practice as someone striving to embrace the discomfort of living with today's tremendous inequalities (Chadwick, 2021). Some of these questions include: What is the relative importance of colonialism as a structural driver of GBV in comparison to other structural drivers, such as gender? How do we produce anticolonial GBV interventions that disrupt the substantial changes wrought by the world's colonial history? Is it possible to return to a precolonial past by maintaining or rejuvenating cultural histories and ideas, or are such efforts poorly aligned with how people live their lives today?

## Comparative Ethnography: Samoa and Peru

To answer these questions, I use a comparative ethnographic approach (Simmons & Smith, 2019) to explore opportunities for GBV prevention through an analysis of how the EVE Project progressed in two Indigenous communities: Samoa and Amantani island in Peru. The different colonial histories marked two very different trajectories for the project. I explore how different colonial histories have respectively shaped community-based resources for challenging GBV and the different strengths, opportunities and pathways that arise for working in partnership with communities to decolonize GBV prevention. Aligned with what Indigenous scholars have referred to as moving forward into the past (Hau‘ofa, 2008), I start with a brief history of colonialism in the two countries, followed by a description of the EVE Project in each setting.

### The EVE Project in Samoa—E le Sauā le Alofa (Love Shouldn’t Hurt)

Samoa, historically referred to as Western Samoa to differentiate it from its American Samoan cousins to the East, was granted independence from New Zealand in 1962. Located in the Polynesian region of the Pacific Ocean, Samoa is in close physical proximity to American Samoa, Fiji and Tonga, with an Indigenous culture that is over 3,000 years old. Samoa has maintained a unique combination of traditional culture and social structures against outside influences and community life remains defined by both the *aiga potopoto* (extended family or clan) and *nu’u* (village). Village councils are organized according to a local system of governance known as the *fa’amatai*, where each family has a hierarchy of *matai* (high chiefs), the most senior of which usually represents *aiga* (family) interests on the council. While *matai* titles are mainly bestowed upon men, women have held *matai* titles since before western European colonialization. Women *matai* numbers are increasing slowly as more and more women take up public leadership roles (Lilomaiava-Doktor, 2020).

While early contact was made between Europeans and the Samoan Islands in the early eighteenth century, it wasn’t until missionaries and traders arrived in the 1830s that the society was transformed dramatically by these relationships. Throughout the nineteenth century, English, American and German forces positioned themselves within the Samoan economy, buying land and profiting from the export of copra and coconut oil (Meleisea, 1987). However, the arrival of the missionaries marked a critical change in social and cultural arrangements. Christianity brought many new conceptual ideas to Samoa and is often spoken of as a fundamental part of the cultural landscape. The Constitution of 1962 states that Samoa is “a Christian nation founded on God the Father, the Son and the Holy Spirit” (Government of Samoa, 1962). Despite its colonial undertones, the arrival of the missionaries is an event that is widely celebrated today, with large community events held in Savai’i to celebrate the arrival of John Williams and the London Missionary Society.

It is difficult, if not impossible, to separate Christianity from the ideas of the colonial government first imposed by Germany over the Western islands of Samoa in 1900. Malama Meleisea's historical account of the colonial period shows how Christianity and Christian values were deeply embedded in the country's governance (Meleisea, 1987). Dr Wilhelm Solf, governor of Western Samoa in 1900, said to a meeting of Samoan chiefs that his government's intention was to “respect your old traditions as far as these are not against the laws of Christianity” (p.47). The Christianization of the country was not one of force but achieved progressively through what many historians have referred to as a peaceful process of deliberation by the Samoan people (Robson, 2010). Some scholars have understood the relatively easy adoption of Christianity as arising from similarities between the traditional *matai* system and Christian beliefs and respect for the male *matai* of the early missionaries (Williams, 1984). However, Christianity has long been considered one of the cultural foundations contributing to wide-spread acceptance of GBV in the Pacific (Kruse, 2021; Nash, 2006; Wendt, 2008). Samoa currently has a prevalence rate estimated at 39.6% of women experiencing physical, sexual, and/or emotional forms of violence by an intimate partner in their lifetime (Samoa Bureau of Statistics, 2020), placing the country well above the global average of one in four women that experience violence globally (Sardinha et al., 2022).

In Samoa, our team from UCL partnered with the Samoa Victim Support Group (SVSG), the National University of Samoa (NUS), and the Samoa Bureau of Statistics to co-develop an intervention to prevent GBV with and for 10 Samoan villages. The project, known locally as *E le Sauā le Alofa* (Love Shouldn’t Hurt) integrated Samoan cultural practices and meanings into the methods used to collect and analyze data and put the locus of control for the project into the hands of 30 village representatives from each of the 10 villages who had an intimate understanding of Samoan cultural practices and protocols. Village representatives were recruited by SVSG as the leading partner organization, a Samoan NGO specializing in emergency response and violence prevention. Together, SVSG, UCL and NUS engaged and trained village representatives in (1) collecting their own data from their local villages about GBV and strategies for its prevention, (2) developing a theory of change based on the data collected, (3) completing a large survey of risk and preventative factors for GBV, and (4) co-designing and piloting an intervention with village representatives drawing on evidence from the Global South about what works to prevent GBV in community settings (Lowe et al., 2023; Mannell et al., 2021).

### The EVE Project in Amantaní, Peru—Allyn Warmi (Women's Wellbeing Project)

Amantaní is an island located in the Peruvian highlands of Lake Titicaca, and the ancestral lands of a Quechua-speaking Indigenous community descending from the pre-Colombian Amerindians (Sandoval et al., 2013). During the Spanish colonial period in the sixteenth century, the Indigenous Quechua and Aymara people of Amantaní, like many other communities in the region, were subjected to forced labor systems such as *encomienda* and *mita*, which disrupted their traditional ways of life and economic practices (Salomon, 1988). The islanders were compelled to work in mines and on plantations, leading to a decline in their autonomy and a shift in their socio-economic structures (Villamarin, 1985). Despite these huge injustices, the inhabitants of Amantaní managed to preserve much of their cultural heritage, including their language, agricultural practices, and communal traditions. Over the centuries, islanders have maintained a distinct identity, characterized by a strong sense of community and a deep connection to their ancestral lands (Starn, 1992). Today, Amantaní is known for its vibrant culture and traditional textile arts, which attract tourists seeking to experience its unique heritage (van den Berghe, 1992).

Our team from UCL partnered with Hampi Consultores de Salud, a small consulting company who had previous experience working with the local communities in Amantaní and had an existing network on the island. First, we completed a needs assessment of the island and services related to GBV in the adjacent city of Puno. This was meant to help identify potential partner organizations that could help provide support for women identified as experiencing GBV as part of the project. Instead, the needs assessment raised several challenges. There were virtually no services available for women from Amantaní that were tailored to their needs as Indigenous communities, no services available in Quechua, and many had a history of overtly discriminatory behaviors against Indigenous populations (Calderon et al., 2023).

We therefore decided to work directly with a group of 10 women and a separate group of 10 men from each administrative district of the island. We created within-project support systems for women who may be experiencing violence, including having a trained psychologist on the team who could provide advice to the research team and psychological support to participants. In most instances, our team psychologist traveled to Amantaní with the research team and helped collect data directly. We also decided not to collect quantitative survey data nor to train our participants to be peer researchers, as we had done in Samoa. Participants expressed discomfort at asking other people in their community about violence, and we felt that without safety mechanisms in place, doing so create risks for women that outweighed the benefits of any data arising from the study. We refocused the study around in-depth qualitative interviews and six strength-based workshops on topics selected by the participants drawing on *buen vivir* (*Sumac Kawsay* in Ecuadorian *kichwa*) as a Latin American framework (Mukerji et al., 2025), which broadly refers to life in harmony with nature and in balance with the cosmos (Tapia Vega et al., 2020).

## Methods

As part of the EVE Project, the research team collected ethnographic data in the form of detailed field notes from both Amantaní and Samoa. In Amantaní, these field notes were recorded by Dr Laura Brown, a Senior Research Fellow at UCL, who traveled to Amantaní in November 2022 and worked closely with the research team in Puno for two years remotely between November 2021 and October 2023. In Samoa, detailed field notes were recorded by me as part of my relocation to Samoa, where I have lived and worked as a visiting researcher at the National University of Samoa since October 2022.

I have used comparative ethnography (Simmons & Smith, 2019) to analyze the field notes collected by myself and Dr Brown, which allows for a systematic comparison of our data from both Samoa and Amantaní island. This provides for a broader understanding of GBV prevention in these two communities by contrasting the ways these different groups understood and addressed GBV prevention as part of the project. Comparative ethnography has previously been used to explore how various cultures perceive and manage health and illness, highlighting significant differences and similarities in medical practices and health beliefs (Langwick, 2008). It has also been used to provide insights into the diverse ways communities construct gender roles and social hierarchies, demonstrating both universal patterns and culturally specific variations (McCallum, 2005). By comparing ethnographic cases, researchers can develop more comprehensive theories that account for the complexity of human societies (Gottlieb, 2004).

Our team at UCL (as my primary academic affiliation) played a coordinating role in the project, developing agendas and materials for workshops. I refer to the project team throughout the paper as the group of practitioners and researchers who implemented the project, including our team at UCL, NUS, and SVSG in Samoa, and Hampi Consultores de Salud in Peru. However, in reality, the EVE Project was largely directed by the community representatives, and we deferred to their decisions on how the project should progress. While I speak in the first person in this paper and take full responsibility for the thoughts and ideas shared, my perspective has been informed by countless others, including the community representatives, my mentors, Indigenous leaders, collaborators, and colleagues at NUS, SVSG, Hampi, and UCL.

## Epistemic Entry-Points Into Decoloniality

I present three “epistemic entry-points” that have shaped our unique trajectory as a project and offer new insights for the diversification of knowledge systems used in GBV research and practice as a move towards decoloniality (Kovach, 2015; Smith, 2013). I refer to these as epistemic entry-points to emphasize the potential for alternative forms of knowledge about GBV in contrast to the dominant discourses currently used in GBV research.

### Epistemic Entry-Point 1: Coloniality of GBV Versus the Patriarchal Narrative

The EVE Project started with the aim of co-designing local strategies with Indigenous communities to prevent GBV using evidence on violence prevention from other settings in the Global South. While the “evidence” we used to explore violence prevention with communities in Amantaní and Samoa looked very different due to the different trajectories of the project, the patriarchal narrative was largely the same—GBV was understood by the project team as a means for men to maintain dominance and control over women—consistent with most interventions to address gender-based violence in the Global South. In GBV prevention research, patriarchy is upheld as an essential risk factor that interventions need to address to be effective through challenging individual social norms of gender inequality and the broader acceptance of GBV by communities (Jewkes et al., 2015; Michau et al., 2015).

In both settings, local communities contested this understanding of gender inequalities as driving GBV. Gendered roles were seen as complementary in both Samoa and Peru, rather than reflecting a dominance of men over women. In Amantaní, both men and women talked about how they made decisions together and often worked together to build homes for their neighbors following community principles of reciprocity and collective benefit. In Samoa, village representatives from different communities spoke openly about the absence of female *matai,* not as an indication of women's lack of political decision-making power in communities, but as evidence that women's power and control over community decision-making took place elsewhere—through the women's committee in villages or by influencing the decisions of their husbands who were *matai*. In both settings, communities opposed the idea of men having more power than women, arguing that both men and women had specific roles, and that these roles complemented each other.

The complementarity and transferability of gender roles is widely cited in literature written by and about Indigenous communities in the Pacific and Peruvian Andes (Besnier & Alexeyeff, 2014; European Farran et al., 2016). However, this comes into conflict with dominant discourses used by GBV researchers where women's experience of violence by male partners is almost always understood as the result of hierarchies of power between men and women. This raises difficult questions about what to focus on in GBV interventions if we do not rely on power hierarchies and gender inequalities as a mechanism by which such interventions work?

The conflict raised by an Indigenous understanding of gender as complementary, relational, and unembodied versus the discourse of power hierarchies between men and women used in GBV research becomes two different and equally valid ways of understanding gender relations. High-intensity patriarchy and its contribution to GBV is not erased in this debate but understood as resulting from colonialism and Christian understandings of gender relationships (Gamlin, 2020). This shifts attention to the story of colonialism as a risk factor for GBV, taking the onus off the gender norms held by community members, and situating the problem in its structural determinants. However, as described below, this was not as easy in practice and the context largely determined what we were able to do.

### Epistemic Entry-Point 2: Christian Versus Indigenous Narratives of GBV

Blaming the high-intensity form of patriarchy brought by colonialism as a direct cause of GBV was largely rejected by communities in Samoa. As highlighted in the background to this paper, the most significant socio-cultural change was the introduction of Christianity, and this has been widely embraced as part of *fa’asamoa* (the Samoan way). There is very little space for any critique of Christianity in Samoan society, raising challenges for a critique of high-intensity patriarchy as a colonial practice when patriarchy is widely condoned and sanctioned as part of the Bible, and is therefore perceived to be the word of God. For example:

Wives, submit to your own husbands, as to the Lord. For the husband is the head of the wife even as Christ is the head of the church, his body, and is himself its Saviour. Now as the church submits to Christ, so also wives should submit in everything to their husbands. (Ephesians 5:22–24)

Biblical texts, such as this, are impossible to openly contest or contradict in Samoan society because of their sacrosanctity. However, in practice Biblical texts have many layers of interpretation and meaning that are often explored through comparison with other Biblical texts by *faifeau* (pastors). This provides an entry-point for challenging the patriarchal nature of Biblical texts when done by the right person. A skilled facilitator as part of the EVE Project, who happened to be the daughter of a *faifeau,* knew this implicitly and was able to challenge village representatives’ conceptualization of the patriarchal relationship as condoned and revered by God. She did so by encouraging them to read beyond the quote from Ephesians 5:22–24, which a few sentences later, turns into an alternative message of love and equality between men and women:

…love your wives as Christ loved the church and gave himself up for her. (Ephesians 5:25)

…love their wives as their own bodies. (Ephesians 5:27)

Ah Siu-Maliko's (2019) comprehensive study of Biblical texts and how they can be used to oppose GBV in the Samoan context also provided a foundation for our pilot intervention. However, this has its own limitations. Reinterpreting Biblical texts using alternative or less well-known texts from the Bible does not directly challenge high-intensity patriarchy nor does it ground it in its colonial origins. In discussing this with an Indigenous Samoan academic and colleague, she greatly opposed our approach saying that the Bible was not Indigenous, quoting Audre Lorde's argument that “the masters tools will never dismantle the master's house” (2003). However, within the constraints of Samoa's highly Christian society, using the master's toolbox was the only discourse available to us as a project team.

In comparison, the Peruvian case offered a perspective that was less supportive of men's authority over women and more clearly grounded in Indigenous principles of gender complementarity. As a project team, we actively listened for this alternative perspective on gender equality, for example, during the following discussion among women during a workshop on domestic violence:

But I think both women and men fulfil almost the same roles. Of course, men have their roles, and we women do too, right? We cook, work, study, the same as men. Of course, some men don't cook, but others do. I think it depends on each family.

The complementarity of gender roles in Amantaní equally advanced our search for an alternative to gender inequality as an explanation for GBV in Quechua-speaking Indigenous communities like Amantaní. It became evident through ongoing discussions with participants that one of the most prevalent experiences of inequality were the stigma and discrimination they faced as an Indigenous community. While participants rarely spoke of this discrimination or its colonial history as a root cause of violence, they did speak of the widespread discrimination they faced outside of their community:

Of course, our fathers, our mothers, our grandfathers, for example, no longer wanted to teach Quechua, to speak the language, because they said: “Don't speak [Quechua] because you're going to speak the “mote” [highland accent], you'll make a mistake and they'll identify you as an Indian and then they'll make fun of you.” One way to protect you was not to teach you Quechua.

Theoretically, these forms of discrimination have been linked to the perpetration of violence by men (Anyikwa, 2015; Reed et al., 2010; Stueve & O’Donnell, 2008). For men, experiences of discrimination can be demasculinising, and in the machismo cultures of Latin America, this can lead men to use violence against women as a means of reaffirming their “lost” masculinity (Viveros-Vigoya, 2016). Empirically as a project, the realization of the active stigma and discrimination the people of Amantaní were facing changed our approach to the study. It became clear that asking community members about GBV prevalence and risk factors in the way that we had done in Samoa would act as its own form of violence by reaffirming the deficits of the community and thus contributing further to the stigma being used against them (Bryant et al., 2021). We decided instead to focus on community strengths as a means of emphasizing the positives of Indigenous culture as part of our research on GBV prevention drawing on frameworks of *buen vivir* (Gudynas, 2011; Mukerji et al., 2025).

The context influenced our project team's decision-making in different ways in the two settings. The highly religious context of Samoa influenced what could and could not be said about colonialism, which is inherently linked to Christianity in this context. In Peru, using the positive strengths of the community as the foundation of our research became essential against a backdrop of extreme stigma and discrimination outside of the community. As a project team, we were fundamentally guided by a difference in the strength of the two communities to assert their perspective on the project, and our own sense of caution in being the cause of further violence.

### Epistemic Entry-Point 3: An Indigenous Island in a Nation Versus a Nation of Indigenous Islands

An Indigenous mentor of mine asked me why we decided not to apply the same strengths-based approach in Samoa as we had in Amantaní? For me, the answer to this question arises from the inherent strength of a cultural worldview, and again, I see this as rooted in the colonial histories of the two settings.

Amantaní island and its Indigenous population have had a long history of degrading and subversive colonial labor experiences including the *encomienda* system where non-Christian Indigenous peoples were required to perform labor in exchange for a Christian education (Zavala, 1984). This was later transformed into the *mita* system where a rotation of Indigenous labors was put in place for men to work for the Spanish colonial state, primarily in silver mines to fund European wars of expansion (Cole, 1985). This resulted in the collapse of the Indigenous population in Peru due to high rates of mortality and outward migration from Indigenous areas (Carpio & Guerrero, 2021). Today, Peru is a settler state with an Indigenous population that represents 25.7% of the total population. In contrast, despite a period of colonial governance first by the Germans and later by New Zealand, Samoa had no major initiatives by the colonial state regarding indentured labor or slavery. There was a period of instability in the first half of the twentieth century with protests led by an anti-colonial group called the *Mau*, which ultimately led to the country's independence in 1962 (Field, 1984). The country of Samoa remains largely Indigenous.

This history tells us about the extent to which two Indigenous cultures have the ability to shape their own destiny (as a cornerstone of decoloniality (Segato & Monque, 2021)). Amantaní exists as an island within the Peruvian nation under the governance of a government controlled largely by the settler population. While Indigenous communities in Peru have made a certain amount of progress on autonomy (Larrea Burneo, 2023), this exists within a national system that affords this autonomy and can just as easily take it away. In contrast, Samoa is a nation with its own unique governance structure, which merges capitalist interests with principles and practices of good governance drawn from the traditional *fa’amatai* system (Poppelwell & Overton, 2022).

The autonomy of Samoa allowed the EVE Project to ask questions about GBV provided that local cultural values were upheld and respected, including building partnerships with Samoan organizations, using (and reproducing) the Samoan language, and adhering to community protocols of respect and reciprocity. We knew that if the project did not adhere to these unspoken rules, that village representatives would reject the project and ultimately fail to participate in project activities, as had happened in other initiatives (Lim-Bunnin, 2020). In contrast, in Amantaní, the community would not have rejected the project because of the community's history of health education campaigns from the Peruvian state, the work of Christian organizations, and the extractive behavior of industry working with Indigenous communities in the region (García, 2005). Instead, the project team was under an obligation to consider for ourselves whether the research represented a benefit to the communities, in the absence of any consideration by ethical review and government authorities about the power dynamics that may have arisen from the country's particular history of colonialism.

## The Praxis of Decoloniality as Part of GBV Prevention

The project of decoloniality as it has been explained by Mignolo and Walsh (2018) is a process of building an alternative or “otherwise” to coloniality in the form of “interculturality.” Interculturality, put simply, is about diversifying the systems of knowledge that are accepted and utilized as part of global discourses. It is about moving beyond singular narratives of Western progress or “development” toward a more complex and dynamic understanding of the interlinkages that exist between multiple discourses and different forms of knowledge. Decoloniality in relation to GBV therefore becomes about establishing new spaces for different systems of knowledge (i.e., Indigenous) to come into dominant discourses and diversifying the forms of knowledge that are seen as expected or taken for granted. As shown in the case studies presented, diversifying understandings of GBV may mean letting go of assumptions about patriarchy as it has been defined by Western feminists as male dominance over women in all aspects of paid and unpaid labor—a relationship that is then affirmed and reinforced through violence – and offering up a more nuanced story.

For example, Rita Segato's work is useful in diversifying current understandings of GBV (Segato & Monque, 2021). Similar to the foundational work of Oyewumi (2004), Segato and Monque argue that gender relations were fundamentally modified and reshaped by colonial powers into a “high intensity” form of patriarchy that saw divisions between men and women as fundamental to the order of society. While Segato and Monque acknowledge that patriarchy did exist prior to colonialism, they argue that this took a fundamentally different form, which they call “low-intensity patriarchy.” However, for the purposes of colonial administration, colonial authorities needed to ensure that there were clearly defined differences between the public (men's sphere) and the private (women's, domestic) sphere. The move from “low-intensity” to “high-intensity” patriarchy happened in Latin America through the village's engagement with the colonial state—men were called upon to represent their community while, at the same time, emasculated when they travel outside of it. In the Pacific, separation of the domestic and public spheres was largely done by Christian missionaries, who drew clear distinctions between men and women to “save” women from hard manual labor (Jolly, 1989). However, in the process, they also replaced a gendered system that consisted of a more flexible movement between genders, evidenced by the *fa’afafine* gender identity in Samoa, the *mahu* in French Polynesia, and *fakafefine* in Tonga—socially prescribed positions for those who are considered as neither male nor female, predating the arrival of colonial power to the Pacific (Tcherkézoff, 2022).

Drawing on Segato and Monque's work helps to ground discourses of gender as a binary and hierarchical relationship based on male dominance over women, situating these in the colonial past (Connell, 2012). This makes possible a search for an alternative *otherwise* in the way people talk about gender and GBV. The idea is not to seek out a gender-neutral past, which has been highly contested (Jolly, 1989), but to offer a viable alternative to feminist discourses of men using violence as a means of dominating or controlling women (Dobash & Dobash, 1979). This is not to say that patriarchy is not a risk factor for GBV, but rather, the intention is to ground patriarchy in its colonial origins, and in so doing, open up new spaces for different understandings of patriarchy through meaningful community interactions.

## Recommendations for GBV Prevention Research and Practice

As with most fields of research, GBV prevention comes with its own theories, frameworks and assumptions that have shaped and defined research practices and scholarly engagements. Introducing a discussion about decoloniality into the field can be daunting for its subversiveness and perceptions of antagonism. We need to find new ways to contend with debates on decoloniality while also being careful not to throw away the last 25 years of GBV research and activism.

Firstly, we need to diversify our knowledge systems about what gender means for GBV. This has been the main thrust of this paper, and an important epistemological project. In the introduction to this paper, I shared three different theoretical explanations for a country's history of colonialism as a structural factor leading to increased GBV: socio-legal systems (Jolly, 1989; Korieh, 2010; Sheldon, 2018), historical trauma and its psychological impacts (Fanon, 2008; Riley et al., 2022), and the coloniality of gender (Segato & Monque, 2021). The comparative case study of Peru and Samoa shows how this division between explanations is much more slippery and contextual, with different explanations driving high rates of violence in different settings. While historical trauma may indeed be a clear driver of GBV among Quechua speaking communities in Peru given their traumatic history, in Samoa, the high-intensity patriarchal forms brought by the Christian missionaries has played a much more subversive and deeply embedded role in shaping gender relations. Similarly, gender relations put women (and sometimes men) at risk of violence in comparatively different ways across contexts. Discounting a particular narrative of gender as part of GBV interventions can be risky in Indigenous settings where these are not the only understandings of GBV that exist. In other contexts, gender may exclusively be defined in its high intensity formulation without alternatives that bring into question the hegemony of this discourse. The objective must be to search out understandings of gender held locally that disrupt GBV for each context.

Second, we need to start having honest conversations about the power dynamics embedded in the methods widely used for GBV research and the ontological/ epistemological viewpoints they reflect. This point has been widely raised by Indigenous methodologists including Kovach (2015), Smith (2013), Chilisa (2012), Suaalii-Sauni and Fulu-Aiolupotea (2014), but has not fully been adopted as part of discussions about GBV research or prevention interventions. The absence of this discussion is partly due to the high involvement of GBV activists in current debates, which equally should not be undervalued or discarded. It is also the result of social epidemiologists playing a stronger role in GBV research. Epidemiology has strengthened the field of GBV research, creating accountability on the part of nation states for collecting and analyzing GBV data. However, drawing from a postpositivist research paradigm that sees reality as observable and measurable (Denzin, 2010), social epidemiologists rarely ask about the power relations inherent in methods that aim to quantify and evaluate.

Current debates about co-production in GBV research are welcome, however these are also insufficient as a means of decoloniality (Braham et al., 2021; Mannell et al., 2023). There is a difference between inviting potential end users to contribute as equal partners in designing an intervention (which is certainly welcome!) and asking them which methods they would use to do this. There is also a hegemony that comes with suggesting that any community needs a GBV prevention intervention in the first place. Decoloniality requires a step back in our thinking, taking the time required to build meaningful relationships with communities and groups, and to conduct research that they themselves have defined as valuable and needed (Smith, 2013). This raises other important questions about how we define “community” and who has the authority to speak within communities, that are also important to tackle (MacQueen et al., 2001). However, our Peruvian experience shows that doing so does not necessarily mean that we need to abandon the project of preventing GBV in high prevalence communities but instead recognizing the multiple ways in which GBV is reproduced under different forms and often referred to using different names.

Lastly, I suggest the need to integrate new theory-praxis spaces in collaboration with GBV-affected groups. This means talking to those affected by violence about theories of the causes of GBV and its roots in colonialism (and other structural determinants, such as climate changes and neocolonial capitalism). It means mapping the praxis involved in decoloniality with those struggling with violence, to surface local understandings of the relationship between GBV and its structural determinants. For example, colonialism may not be perceived as “bad” or “negative” according to the worldview of all Indigenous peoples, and while we can see it as the negative repercussions it has had, not all groups may see it in the same way. There may be different starting points for disrupting its negative impacts, and what these starting points are, also needs to be defined and shaped in conversation with those affected by it. Decolonial GBV research often emphasizes the intersectionality of oppression, recognizing how colonialism, patriarchy, and other forms of systemic inequality intersect to shape the experiences of violence among different groups (Lugones, 2007). This holistic approach not only enriches the academic understanding of GBV but also contributes to more just and equitable policy and practice outcomes.

## Conclusions

I do think it is both possible and necessary to bring decoloniality theories into GBV research, however it may not be desirable to “decolonize” research. In 2021, Richard Horton argued in *The Lancet* that global health cannot ever be truly decolonized: that an imperial power is almost always replaced by another and that what we may be doing with current decolonization practices (such as bringing down statues or burning books) is actually erasing history rather than revealing its fundamental truths (Horton, 2021). Whether intentional or not, Horton's argument is closely aligned with Indigenous understandings of power within a regenerative system: power cannot be destroyed, only transferred as part of an interconnected and relational system (Yunkaporta, 2019). Given the tendency of global health (and GBV research done under its banner) to obscure history and power from its analyses, drawing attention to the power relations instilled by the past becomes even more important, not less so.

To draw attention to the operation of power in GBV prevention, the case studies from Peru and Samoa demonstrate the importance of diversifying the forms of knowledge (including knowledge about gender relations) that we value for the field. We also need to diversify the methods we use beyond an epidemiological toolbox, to bring new understandings of the structural causes of violence. As suggested by Lokot et al. (2024), this research needs to be conceptualized and directed by affected communities and groups. It is only through local practice and meaning-making that we can begin to understand the multiple ontologies and epistemologies of violence, which have important implications for the success of GBV prevention interventions.

As a first step, our research experience points to the critical need for an Indigenous understanding of GBV and the methods that can be used towards prevention. This was one of the intentions behind the EVE Project, but now that we are reaching the end, I don’t think the project has gone far enough in questioning its ontological and epistemological assumptions. According to the Australian Indigenous academic Tyson Yunkaporta, Indigenous thinking is premised on the idea of a regenerative, interconnected and interrelated system where nothing can be created or destroyed. This has important implications for GBV prevention interventions that try to eliminate violence. An Indigenous approach might focus instead on redirecting the violence into a form that is less harmful and destructive (i.e., rituals, ceremony, sport) (Yunkaporta, 2019). This has implications far beyond Indigenous thought, with similar ideas brought about through engagements with new materialist approaches to GBV (Clark, 2023; Fox & Alldred, 2022), highlighting the broader applicability of efforts to diversify our knowledge beyond dominant discourses about violence and its prevention.

And finally, a word of caution: I think we need to be careful in our efforts to “decolonize” GBV research and should instead be focusing on practices related to decoloniality. We must not erase dynamics of power from our analyses or continue to ignore the importance of colonial relationships in supporting violent behaviors. I agree with Horton and his cautioning of the decolonization agenda. We need embedded research on GBV conducted by Indigenous academics and their allies but could do without the expert-narrative still so prominent in global health.
